# Supplementary material for: Reference genes selection for transcript normalization in kenaf (Hibiscus cannabinus L.) under salinity and drought stress
Source: PeerJ. 2015 Nov 26;3:e1347. doi: 10.7717/peerj.1347 (PMC4671189; doi:10.7717/peerj.1347)
Supplement: Supplemental Information 1 [file peerj-03-1347-s001.pdf]

S1: PCR products of 10 reference genes checked on a 2.0% agarose gel.

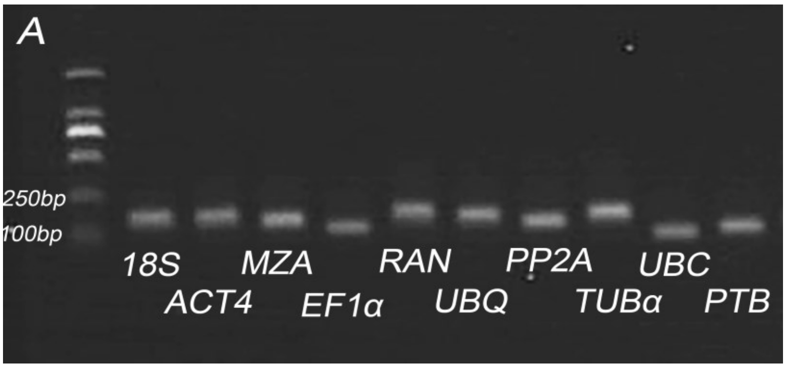

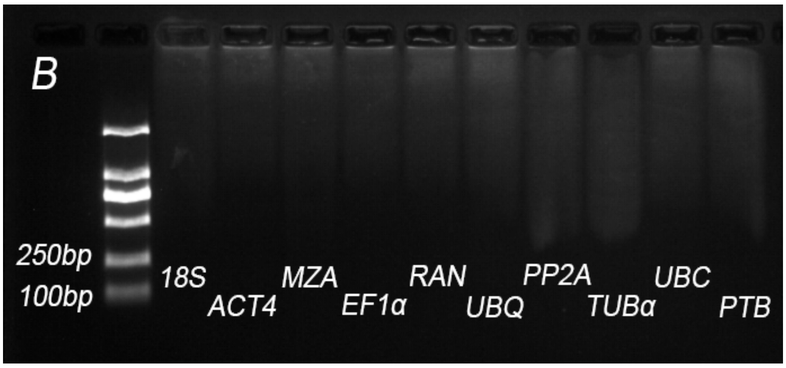

A: DNAse digested RNA subjected to a cDNA synthesis with Reverse Transcriptase.

B: A negative RT reaction whereby DNAse digested RNA is subjected to a mock cDNA synthesis without Reverse Transcriptase.
