# Supplementary figures and images for: Reference genes selection for transcript normalization in kenaf (Hibiscus cannabinus L.) under salinity and drought stress
Source: PeerJ. 2015 Nov 26;3:e1347. doi: 10.7717/peerj.1347 (PMC4671189; doi:10.7717/peerj.1347)

S2: Melting curves of the 10 candidate reference genes tested in this study.

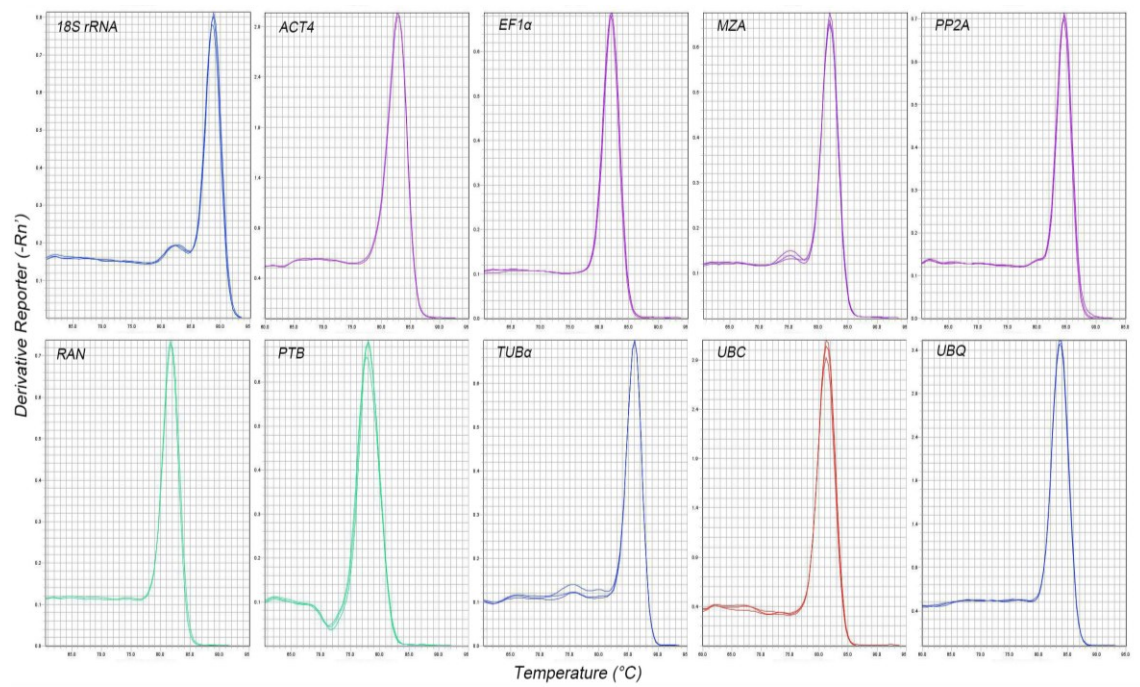

Supplement: Supplemental Information 2 [file peerj-03-1347-s002.pdf]
